# Unravelling the Antibacterial Activity of *Terminalia sericea* Root Bark through a Metabolomic Approach

**DOI:** 10.3390/molecules25163683

**Published:** 2020-08-13

**Authors:** Chinedu P Anokwuru, Sidonie Tankeu, Sandy van Vuuren, Alvaro Viljoen, Isaiah D. I Ramaite, Orazio Taglialatela-Scafati, Sandra Combrinck

**Affiliations:** 1 Department of Chemistry, University of Venda, Private Bag X5050, Thohoyandou 0950, South Africa; anokwuruchi@gmail.com (C.P.A.); Isaiah.Ramaite@univen.ac.za (I.D.I.R.); 2 Department of Pharmaceutical Sciences, Tshwane University of Technology, Private Bag X680, Pretoria 0001, South Africa; tanksido@yahoo.fr (S.T.); viljoenam@tut.ac.za (A.V.); 3 Department of Pharmacy and Pharmacology, Faculty of Health Sciences, University of the Witwatersrand, 7 York Road, Parktown 2193, South Africa; sandy.vanvuuren@wits.ac.za; 4 SAMRC Herbal Drugs Research Unit, Faculty of Science, Tshwane University of Technology, Private Bag X680, Pretoria 0001, South Africa; 5 Department of Pharmacy, University of Naples, Federico II Via D. Montesano 49, 1-80131 Napoli, Italy; scatagli@unina.it

**Keywords:** antibacterial, *Terminalia sericea*, metabolomics, chemical markers, chemometrics, root bark, compounds

## Abstract

*Terminalia sericea* Burch. ex. DC. (Combretaceae) is a popular remedy for the treatment of infectious diseases. It is widely prescribed by traditional healers and sold at informal markets and may be a good candidate for commercialisation. For this to be realised, a thorough phytochemical and bioactivity profile is required to identify constituents that may be associated with the antibacterial activity and hence the quality of raw materials and consumer products. The aim of this study was to explore the phytochemistry and identify the antibacterial constituents of *T. sericea* root bark, using a metabolomic approach. The chemical profiles and antibacterial activities of 42 root bark samples collected from three districts in the Limpopo Province, South Africa, were evaluated. Dichloromethane:methanol (1:1) extracts were analysed using ultraperformance liquid chromatography (UPLC)-mass spectrometry (MS), and chemometric models were constructed from the aligned data. The extracts were tested against *Bacillus cereus* (ATCC 11778), *Staphylococcus epidermidis* (ATCC 12223), *Staphylococcus aureus* (ATCC 25923), *Escherichia coli* (ATCC 8739), *Klebsiella pneumoniae* (ATCC 13883), *Pseudomonas aeruginosa* (ATCC 27853), *Shigella sonnei* (ATCC 9292) and *Salmonella typhimurium* (ATCC 14028), using the minimum inhibition microdilution assay. Nine compounds; sericic acid, sericoside, resveratrol-3-*O*-*β*-rutinoside, ellagic acid, flavogallonic acid dilactone, methyl-flavogallonate, quercetin-3-(2′′-galloylrhamnoside), resveratrol-3-(6′′-galloyl)-*O*-*β*-d-glucopyranoside and arjunetin, were isolated from the root bark. All the compounds, with the exception of sericic acid, sericoside and resveratrol-3-*O*-*β*-rutinoside, were isolated for the first time from the root bark of *T. sericea.* Chemometric analysis revealed clustering that was not population specific, and the presence of three groupings within the samples, characterised by sericic acid, sericoside and an unidentified compound (*m/z* 682/4.66 min), respectively. The crude extracts from different populations displayed varied antibacterial activities against *S. typhimurium* (minimum inhibitory concentrations (MICs) 0.25–1.0 mg/mL), but similar activity towards *Bacillus cereus* (1.0 mg/mL). Several compounds present in the root bark were highly active towards all or most of the pathogens tested, but this activity was not reflected by the chemical profiles of extracts prepared from the individual samples. Among the pure compounds tested, only flavogallonic acid dilactone and methyl-flavogallonate exhibited broad-spectrum activity. A biochemometric analysis indicated that there was no consistent association between the levels of phytochemicals and the activity of the active or non-active extracts. Although it was deduced that the major constituents of *T. sericea* root bark contributed to the chemotypic variation, further investigation of the interactions of compounds present in the root bark may provide antibacterial efficacies not evident when examining compounds singularly. The data reported herein will provide information that is fundamentally important for the development of quality control protocols.

## 1. Introduction

*Terminalia sericea* Burch ex. DC. (Combretaceae) is among the 50 most popular medicinal plants in Africa [1]. 1 It is a small to medium-sized tree of about 5–8 m, although some trees can reach 23 m in height [2]. The tree is widely distributed throughout Africa, particularly in savannah woodland environments [3,4]. *T. sericea* is well known across many cultures and referred to as the silver cluster leaf, “vaalboom” in Afrikaans, “mangwe” in isiNdebele, “moxonono” in Sesotho, “mususu” in chiShona and Tshivenda, “mongonono” in Tswana, “amangwe” in isiZulu, and as “namatipo” or “mpululu” in Nyakusa (Tanzania), “mukenge” in Katima Mulilo (Namibia), and as “nsunsu”, “nkonola”, “kondla” or “mogonono” in Canhane (Mozambique). 

Although decoctions of fruits, leaves and stems are used for medicinal preparations, the root decoction is by far the most utilized part of the plant [4]. Over the past few decades, the benefits of *T. sericea* have transcended African borders and the stem bark has also been sold to the European market [5]. Extracts of the plant, standardised with reference to sericoside (a major constituent), or pure sericoside, are sold for their anti-inflammatory activities, with claimed anti-wrinkle or anti-aging effects [6,7]. In Africa, *T. sericea* root decoctions are administered for the treatment of a range of conditions, such as stomach ailments, diabetes, asthma, and constipation, but the main applications are associated with bacterial infections, including diarrhoea, sexually transmitted diseases, coughs, fever and wound infections [8]. The antibacterial activities of the crude polar organic and aqueous extracts against both Gram-positive and Gram-negative pathogens have been investigated extensively [9,10,11,12,13,14,15,16,17,18,19]. Some studies [20,21] proposed that anolignan b, termilignan b and arjunic acid are the main constituents responsible for the antibacterial activity of the root extract, since they were highly active against several bacterial pathogens (*Bacillus cereus*, *Escherichia coli*, *Klebsiella pneumoniae* and *Staphylococcus aureus*), with minimum inhibitory concentrations (MICs) in the range of 1.9–31 µg/mL. However, these studies considered samples from a single population, thus not accounting for possible chemotypic variation that could lead to correlated variation in the biological activity. 

One of the main challenges encountered in drug development from natural sources is the loss of activity observed in isolated compounds compared to that of the crude extracts. The search for a single highly bioactive compound does not adequately consider the interaction of the intricate and concatenated network of secondary metabolites present in medicinal plants [22]. Antimicrobial activity is a good example in this context, since plants are composed of complex phytochemical pools of compound combinations designed to fight infections, and often molecules isolated from plant sources have exhibited lower antimicrobial activities compared to the whole extracts [23,24]. However, the study of plant extracts remains inherently complex and is often exacerbated by rampant variation due to genetic traits and/or environmental factors that influence the production of secondary metabolites by plants. It is therefore important to have knowledge of the phytochemical variation within a species, and to understand how the variation is linked to the differences in the bioactivity of extracts prepared from individual samples [25]. 

The commercial potential of *T. sericea* as an anti-infective agent has not been fully exploited due to the lack of adequate standardisation and quality control. This study aimed at exploring the phytochemical and antibacterial activity variation of *T. sericea* root bark samples, obtained from different districts in the Limpopo Province of South Africa using a metabolomics approach, to isolate the main root bark constituents and to determine their role in the observed activity. Although methods for isolating metabolites from root bark material of *T. sericea* have previously been reported [26,27,28], details on the isolation procedures are not available, making them difficult to replicate. Previous methods involved several solvent partitioning steps, followed by silica gel chromatographic separation, to isolate the aglycones and glycosides, respectively. In this study, we present a direct column chromatography method, bypassing the initial solvent partitioning step, which accelerated the isolation workflow. 

## 2. Results and Discussion

### 2.1. Untargeted UPLC-MS and Discriminant Analysis 

**Figure 1:**
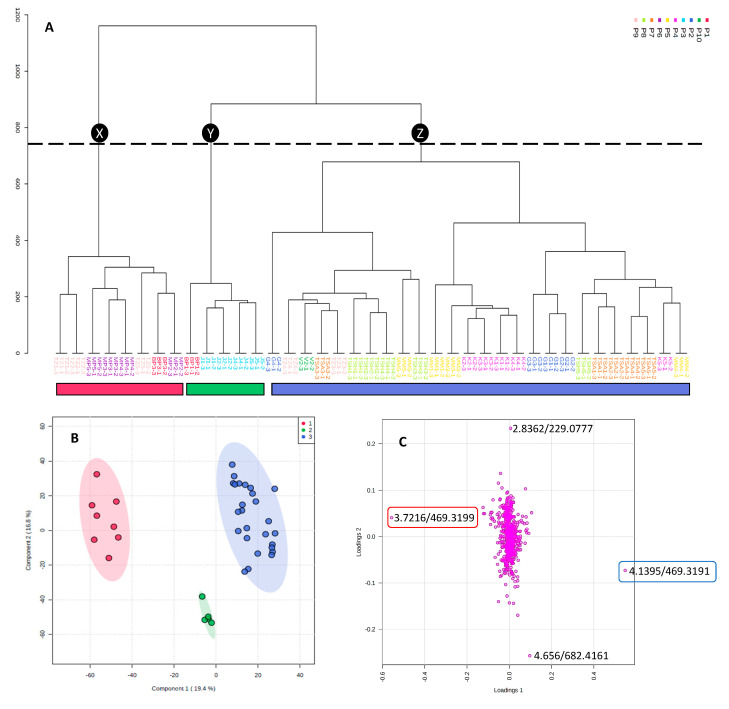
(**A**) Hierarchical cluster analysis dendrogram of the ultra performance liquid chromatography (UPLC)-mass spectrometry (MS) data (N = 39) obtained from Waterberg, Mopani and Vhembe districts of Limpopo Province. Branch X (red): samples from Waterberg and Mopani; branch Y (green): samples from Waterberg and Vhembe; branch Z (blue): samples from Mopani and Vhembe districts. (**B**) Partial least square discriminant analysis (PLS-DA) of clusters from the dendrogram. (**C**) Loadings scores plot obtained from the PLS-DA analysis. Compound indicated by red rectangle contributed to the clustering of the Red (X) group, while compound in blue rectangle contributed to the clustering of the Blue group (Z). These two compounds are the first two variables indicated by the variable importance for project (VIP) plot.

A hierarchical cluster analysis (HCA) was conducted from a constructed principal component analysis (PCA) model (plots not shown), characterised by five principal components, which explained 62% of the variation in the data. The HCA was used to investigate the chemical variation (LC-MS data) within the samples. The dendrogram obtained (Figure 1A) indicated three main branches (X, Y and Z). The first branch X (red) consists of samples from Waterberg (P1, P6) and Mopani (P9) districts. The second branch Y (green) comprises samples from Waterberg (P1) and Vhembe (P3) districts, while samples from Mopani (P4) and Vhembe (P4, P5, P7, P8) districts form the third branch Z. The largest variation was observed for population P9 (samples distributed within the first and third branches), while the least variation was observed for population P3 (second branch). The exact location of samples from P4 is unknown, since the samples were purchased from an informal “*muthi*” market in Thohoyandou. Clustering of these samples (K1–K5) with samples from population P5 (Vhembe District) strongly suggests similar chemistry and hence the possible origin of the market samples. 

The three main branches observed from the dendrogram with localities distributed between the three groups, resulted in the identification of markers for each group. Therefore, partial least squares-discriminant analysis (PLS-DA; Figure 1B) was carried out to identify the compounds contributing to the clustering observed in the dendrogram. A four-component PLS-DA model was constructed, indicating 89.2% variation with an accuracy of 97.2%. Groups Red (X), Green (Y) and Blue (Z) separated along PC1, with group Red (X) and Green (Y) distributed on the negative PC1 and group Blue (Z) on the positive PC1. In addition, a separation can be observed along PC2 with the Red (X) and Blue (Z) groups mainly distributed on the positive PC2, while all samples from the Green (Y) group are situated on the negative PC2. Variable importance for project (VIP) scores were used to identify the compounds contributing the most to the discrimination observed in the loadings plot (Figure 1C). Compound *m/z* 469 (Rt 3.72 min) contributed to the clustering of samples corresponding to group Red (X) of the PLS-DA, while a compound corresponding to the molecular ion *m/z* 469 (Rt 4.14 min) contributed to the clustering of samples in group Blue (Z). The molecular ion *m/z* 682 (Rt 4.66 min) contributed to the clustering of samples corresponding to group Green (Y). The chemometric analysis indicated that samples from Waterberg district are characterised by higher concentrations of compound *m/z* 469 (Rt 3.72 min), while samples from Vhembe district contain a higher concentration of compound *m/z* 469 (Rt 4.14 min). Samples in group Red (X) and Blue (Z) contain high concentrations of compound *m/z* 299 (Rt 2.84 min), while samples in group Green (Y) have low concentrations. The chemical variability of the samples is further illustrated by the chemical profiles (Figure S1) of representative samples from different populations. This is the first study to explore the variation within natural samples of *T. sericiea* from different geographical locations.

### 2.2. Isolation and Identification of Chemical Constituents 

**Figure 2:**
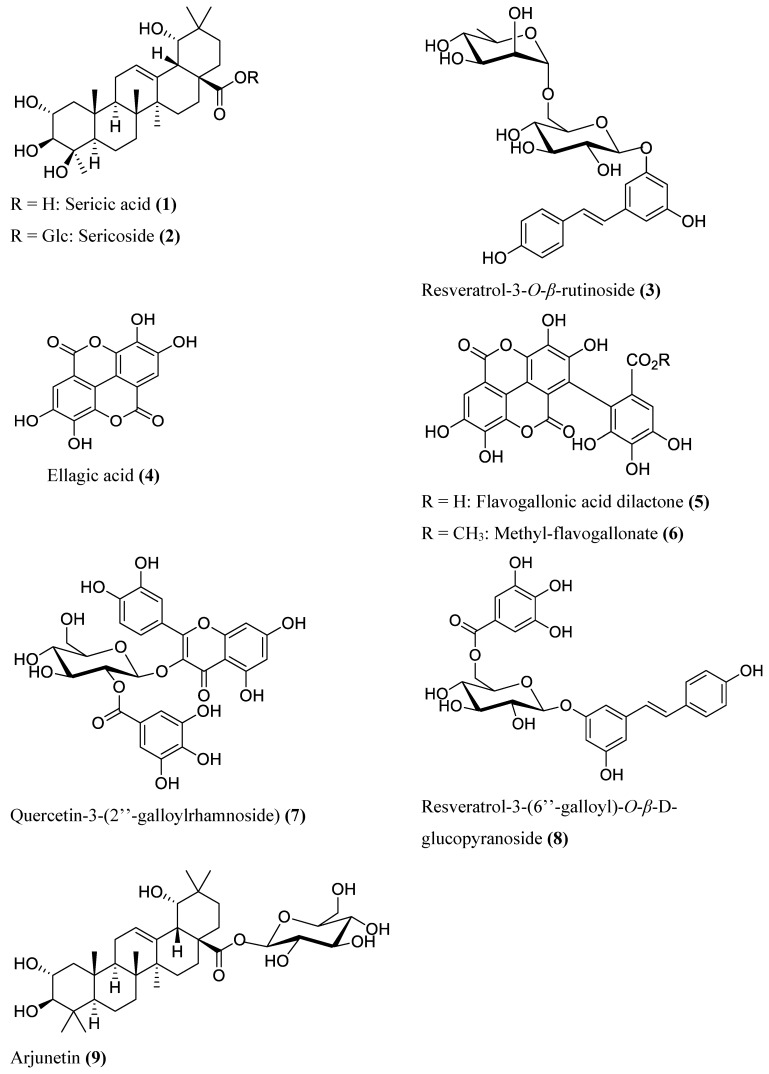
Compounds (**1**–**9**) isolated from dichloromethane:methanol crude extract of *T. sericea* root bark.

During isolation, the major compounds, as well as the active compounds in the antibacterial assay, as revealed through bioassay-guided fraction, were targeted (Figure 2) and are discussed here collectively. The chemical profiles (Figure S1) suggested that compounds yielding fragment ions at *m/z* 229 (Rt 2.83 min), *m/z* 469 (Rt 3.72 min) and *m/z* 469 (Rt 4.14 min) were the major constituents of the *T. sericea* root bark extract, with the last two as marker compounds responsible for the chemical variations observed in the loadings plot (Figure 1C). These three compounds were isolated chromatographically and identified by NMR analysis. 

The compound yielding a fragment at *m/z* 469 (Rt 3.72 min) was obtained as white crystals from Fraction 1 and identified as sericic acid (**1**) on the basis of its spectral data, corresponding to those reported in literature [26,29,30,31]. The molecular ion (*m/z* 527.336) obtained from the high resolution-electrospray ionisation-mass spectrometry (HR-ESI-MS) is consistent with the formula C_30_H_48_O_6_Na [M + Na]^+^ (calculated mass 527.334). The fragment at *m/z* 469 corresponds to the loss of two OH-moieties from the molecule. Marker signals in the ^1^H-NMR spectrum for the oleanane-type triterpenoid structure are the olefin (t, δ_H_ 5.34), hydroxymethylene (dd, δ_H_ 4.05, 3.42), hydroxymethine (ddd, δ_H_ 3.80, 3.32, 3.04) and methyl singlet (δ_H_ 1.29, 1.23, 0.97, 0.96, 0.93, 0.74) protons (Figure S2). 

The compound yielding a fragment at *m/z* 469 (Rt 4.14 min) was obtained as a white solid from Fraction 2 and identified as sericoside (**2**). Spectral data obtained were consistent with those reported in the literature [26,32,33]. The presence of an anomeric proton at δ_H_ 5.24 and carbon signals at δ_C_ 94.6, 78.2, 77.2 and 69.9 (Figure S3) support the presence of the sugar moiety. The molecular ion (*m/z* 689.389) is consistent with the molecular formula C_36_H_58_O_11_Na [M + Na]^+^ for sericoside.

The compound yielding a fragment at *m/z* 229 (Rt 2.83 min) was identified as 3′,5′,4–trihydroxy-resveratrol-3-*O*-*β*-rutinoside (**3**). It was obtained as a brown solid from Fraction 2. The molecular ion [M + H]^+^ (*m/z* 537.336) obtained from HR-ESI-MS is consistent with the formula C_26_H_32_O_12_. The ^1^H and ^13^C spectral data (Figure S4) obtained corresponded to that reported in literature [27,28,34]. 

Ellagic acid (**4**) was isolated as a brown amorphous solid. The molecular ion [M − H]^−^ *m/z* 301 obtained using UPLC-MS/MS is consistent with the molecular formula C_14_H_6_O_8_. The ^1^H spectrum showed a singlet at δ_H_ 7.45, while the ^13^C NMR spectrum showed seven aromatic signals at δ_C_ 107, 110, 112, 136, 140, 148, 159 (Figure S5), which are consistent with the spectral data reported in literature for ellagic acid [35,36].

Flavogallonic acid dilactone (**5**) was isolated as a yellow amorphous powder. Two singlet signals at δ_H_ 7.15 and 7.45 were evident in the ^1^H NMR spectrum (Figure S6A). The ^13^C-NMR signals were in agreement with literature data [37,38] and, in particular, signals at δ_C_ 160.3 and 158.8 (Figure S6C) indicated two lactonised carbonyl carbons. Methyl-flavogallonate (**6**) was isolated as a yellow amorphous powder. The ^1^H and ^13^C-NMR spectral data were similar to those of flavogallonic acid dilactone, but the ^1^H-NMR (Supplementary material Figure S6B) indicated the presence of an additional methyl group. The compound identity was further confirmed when the fragmentation ions (*m/z* 451, 433) obtained from UPLC-MS/MS were compared to literature [39]. This compound was first isolated from the leaves of *Terminalia myriocarpa* [40]. 

Resveratrol 3-(6′′-galloyl)-*O-β*-d-glucopyranoside (**7**) was isolated as a brown amorphous powder. The ^1^H and ^13^C NMR spectral data (Figure S7) mirrored those reported in literature [41]. The aromatic protons at δ_H_ 7.27 (d, *J* = 8.4 Hz), 6.76 (d, *J* = 8.6 Hz), 6.72 (brs), 6.65 (brs), 6.48 (brs) and olefin protons at 6.95 (d, *J* = 16.3 Hz) and 6.86 (d, *J* = 16.3 Hz) are consistent with the ^1^H NMR signals of the resveratrol moiety. The galloyl unit displayed a singlet proton at δ_H_ 7.12 and a carboxylic carbon at δ_C_ 166.9. The protons at δ_H_ 4.65 (dd, *J* = 12.0, 2.1 Hz) and 4.45 (dd, *J* = 12.0, 5.3 Hz) are characteristic of the methylene proton (H-6′′) of the sugar moiety.

Quercetin-3-(2′′-galloylrhamnoside) (**8**) was isolated as a brown amorphous powder. Its ^1^H and ^13^C NMR spectra (Figure S8A & B) were identical to those in literature [42]. Also, the fragmentation ions (*m/z* 599, 447, 300) (Figure S8C) obtained from the UPLC-MS/MS [M − H]^−^ were in agreement with the structure and literature data [43].

Finally, arjunetin (9) (*m/z* 695) was obtained as a white solid, identified on the basis of MS peaks and NMR spectral data (Figure S9A,B) that corresponded to literature data [44]. The fragmentation ion *m/z* 487 (Figure S9C) obtained from UPLC-MS/MS further confirms that the compound with *m/z* 695 is a glycoside of arjunic acid.

All the compounds, with the exception of sericic acid, sericoside and resveratrol-3-*O*-*β*-rutinoside, were isolated for the first time from the root bark of *T. sericea*. Sericic acid was identified as a marker for branch (X), while sericoside was identified as the marker for branch (Z) (Figure 1A). Although the marker for branch (Y) was not isolated in this study, the molecular ion (*m/z* 682) and retention time (4.66 min) is characteristic of a triterpenoid glycoside. The concentration of resveratrol-3-*O*-*β*-rutinoside was high in samples found in branches (X) and (Z), but low in samples in branch (Y).

### 2.3. Antibacterial Constituents of T. sericea Root Bark

The isolated compounds (with the exception of resveratrol-3-(6″-galloyl)-*O*-*β*-d-glucopyranoside **8** due to insufficient amount yield), crude extract and fractions were tested to determine their antibacterial activities. The crude extract and Fraction 1 displayed moderate activity against *B. cereus* with a minimum inhibitory concentration (MIC) of 0.29 mg/mL (Table 1). In contrast, Fraction 2 displayed poor activity at the tested concentrations, and, accordingly, its main constituents, sericoside and resveratrol- 3-*O*-*β*-rutinoside, were considered inactive against most of the pathogens. Sericic acid (**1**), however, displayed antibacterial activity towards all of the pathogens, except *S. typhi*, with the lowest MIC value against *S. sonnei* (0.29 mg/mL) These results encouraged further purification of Fraction 1 to identify compounds responsible for the activities against *S. typhi* (since sericic acid, the major constituent of Fraction 1 was not active against this pathogen). Compounds displaying antibacterial activity against *S. typhi* were isolated through bioassay-guided fractionation.

Ellagic acid (**4**), flavogallonic acid dilactone (**5**), methyl-flavogallonate (**6**), quercetin-3-(2′′- galloylrhamnoside) (**7**), and arjunetin (a glycoside of arjunic acid) (**9**) were isolated as the active compounds, with the lowest MIC value obtained for (**7**) as 0.05 mg/mL against *B. cereus*. Surprisingly, bioassay-guided fractionation did not reveal anolignan b, termilignan b and arjunic acid, previously associated with antibacterial activity [20,21] and these were therefore not isolated during this part of the study. Among all the compounds tested, flavogallonic acid dilactone (**5**) and methyl- flavogallonate (**6**) yielded broad-spectrum activity. In previous studies, ellagic acid displayed antibacterial activities against *S. aureus* (MIC value 0.13 mg/mL) and *P. aeruginosa* (MIC value 0.25 mg/mL) [45]. Arjunetin isolated from the bark of *T. arjuna*, displayed antibacterial activity against *S. epidermidis* with an MIC of 0.13 mg/mL, but was not active against *S. aureus* (MIC > 0.5 mg/mL) or *P. aeruginosa* (MIC > 0.5 mg/mL) [44]. Our study is the first to report the antibacterial activities of isolated compounds of *T. sericea* root bark against a wide range of pathogens.

The antibacterial activities of the isolated compounds (Table 1) provide new information. Eldeen and colleagues [20,21] reported that anolignan b, termilignan b (lignans) and arjunic acid (triterpenoid) are the major antibacterial constituents of *T. sericea* root bark. However, our study suggests that the ellagitannins also play a role in the antibacterial activities. Remarkably, although resveratrol-3-*O*-*β*-rutinoside and sericoside are major root metabolites, they do not seem to contribute directly to the antibacterial activity of the root bark.

### 2.4. Biochemometric Analysis

After establishing the chemical variation within the samples collected from different localities, the next step was to evaluate the effect of this chemical variability by comparing the antibacterial activities of the crude extracts. *Bacillus cereus*, the most susceptible pathogen to the crude extract, and *S. typhi*, resistant to sericic acid (**1**), were considered for this study. Since a good model would involve variability in the antibacterial activities, *S. typhi* was selected based on the chemical variability of sericic acid in the samples. It was expected that samples with a high sericic acid content would have low activity against *S. typhi*. Crude extracts of the 42 samples were tested against these pathogens (Table 2) and virtually all extracts yielded the same MIC value (1.0 mg/mL) against *B. cereus*. Samples from P1.2, P1.3, P2.1, P2.2, P3 and P9.5 displayed higher activities (MIC 0.25 mg/mL) towards *S. typhi*. The variation in antibacterial activities of the samples against *S. typhi* demonstrates the importance of evaluating antibacterial activities of plant samples from different localities to account for possible chemical variations. Since there were differences in the MICs obtained for *S. typhi*, these results were used to conduct a biochemometric analysis to identify the specific compounds (and the combination thereof) that exert activity against this pathogen.

Consideration of the MICs for the crude extract (Table 1) and samples from different populations (Table 2) indicates variations in activities. The crude extract displayed a higher activity against *B. cereus* (MIC value 0.29 mg/mL) than the individual extracts from different populations (MICs 1.0 mg/mL). Conversely, the samples from the individual populations displayed higher activities towards *S. typhi* (MICs 0.25–1.0 mg/mL) compared to the crude extract (MIC 1.5 mg/mL). The crude extract was a composite of samples from different populations, which may have resulted in a different balance of the major constituents. This result highlights the fact that the relative proportions of the secondary metabolites have a significant impact on the activity.

**Figure 3:**
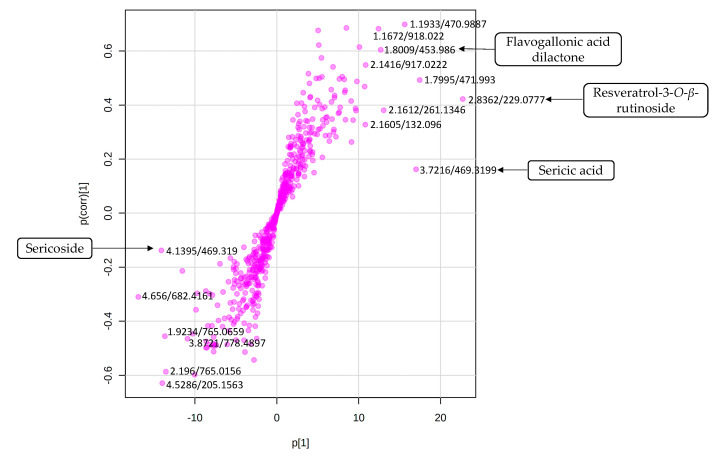
S-plot indicating biomarkers (retention time/molecular ion *m/z*) for the activity against *S. typhi.*

**Figure 4:**
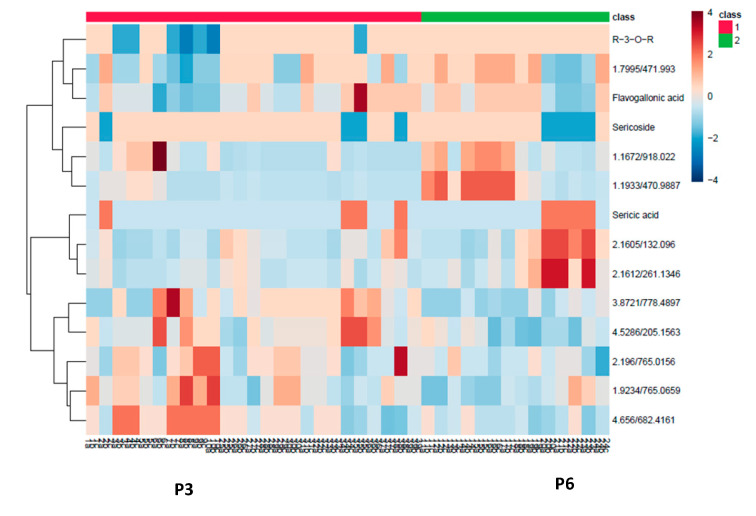
Heatmap of 15 VIP peaks in 39 samples of *T. sericea* root bark tested against *S. typhi*. R-3-*O*-R: resveratrol-3-*O*-*β*-rutinoside.

The biochemometric analysis was carried out to establish the link between the chemotypic variation and the antibacterial activity. Samples were classified into two groups according to their antibacterial activities. Samples with MICs < 1 were grouped as active and samples with MICs values ≥ 1 were grouped as non-active. An orthogonal projection to latent structures-discriminant analysis (OPLS-DA) model was constructed to evaluate the relationship between chemistry and bioactivity. The five-component model obtained for the two preassigned groups (active and non-active) indicated a 54% variation (R^2^), R^2^Y of 99.28% with prediction ability (Q^2^) of 98.6% (Figure S10). Four orthogonal and one predictive latent variable were used. A 20-permeation test is automatically done by the software. The active samples (Red) were distributed on the negative T score 1 and the non-active samples (Green) were clustered along positive T score 1 (Figure S10). Compounds responsible for the discrimination of the two classes were extrapolated from the S-plot generated from the model (Figure 3). However, the selection of compounds contributing significantly to the separation was carried out using a VIP plot (Figure S11). A heat map of the most important compounds was generated to visualise their concentrations in all the samples (Figure 4). In the S-plot, sericoside was found to correspond to the active class, while sericic acid, resveratrol-3-*O*-*β*-rutinoside and flavogallonic acid dilactone were associated with the less active class.

In the heatmap (Figure 4), resveratrol-3-*O*-*β*-rutinoside (2.84/299) was present in several Class 1 and Class 2 samples in moderate concentrations. Similarly, sericoside (4.14/469) was present in several samples in both classes. The concentration of sericic acid (3.72/469) was very low in many samples of both classes. In general, there was no consistent association between phytochemicals and classes. However, when specific populations in each class were considered, association was established. Samples from population P3 (Class 1) were associated with low concentrations of resveratrol-3-*O*-*β*-rutinoside, flavogallonic acid dilactone (1.80/453), sericic acid and the unidentified compound corresponding to the molecular ion *m/z* 471 (Rt 1.79 min). However, these samples were associated with moderate concentrations of compounds corresponding to the molecular ions *m/z* 765 (Rt 2.19 min), *m/z* 765 (Rt 1.92 min) and *m/z* 682 (Rt 4.66 min).

In the inactive Class 2, samples from population P6 were associated with low concentrations of flavogallonic acid dilactone, sericoside and a compound corresponding to the molecular ion *m/z* 471 (Rt 1.79 min), but were high in sericic acid and a compound corresponding to the molecular ion *m/z* 132 (Rt 2.16 min). Both populations displayed low concentrations of flavogallonic acid dilactone, an active compound against *S. typhi*. Therefore, the variation could not have been due to flavogallonic acid dilactone. Inspection revealed that the activity could be due to *m/z* 765 (Rt 2.19 min), *m/z* 765 (Rt 1.92 min) and *m/z* 682 (Rt 4.66 min). However, the retention time of *m/z* 682 suggests a saponin similar to sericoside, which was not active. These compounds may not contribute to the variation observed, since the concentrations of these compounds were not high in other samples. It is important to note that population P3 displayed the highest activity (average MIC 0.25 mg/mL) when tested against *S. typhi* and contained low concentrations of sericic acid and resveratrol-3-*O*-*β*-rutinoside, while P6 contained high concentrations of sericic acid. We suggest that the major constituents, though not associated strongly with any of the classes, contributed to the variation observed. This position is further supported by the activities of the extracts against *B. cereus* that indicated no variation in antibacterial activities, probably since sericic acid was also active along with the other isolated compounds. It is possible that the higher the concentration of the inactive metabolites in the crude extract, the lower the relative proportion of the active compounds, rendering the extract inactive. To confirm the negative contribution of sericoside and resveratrol-3-*O*-*β*-rutinoside to the antibacterial activities of *T. sericea* root bark, synergistic studies of the active and non-active compounds are required.

**Figure 5:**
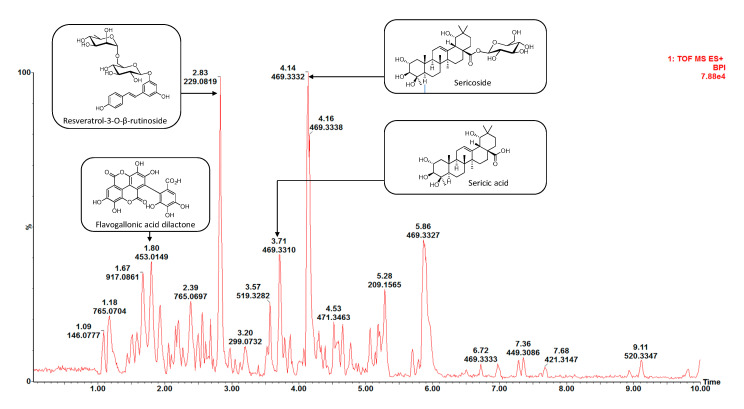
UPLC-MS chromatogram of dichloromethane:methanol crude extract of *T. sericea* root bark indicating bioactive compounds and chemical markers.

A revisit of the chemical profile (Figure 5) suggests that compounds present between retention times 1 and 3 min are ellagitannins and lignans, while compounds that eluted between 3 and 6 min are triterpenoids and saponins. Therefore, it can be concluded that active Class 1 samples (Figure 3) contain mainly triterpenes and saponins, while less active Class 2 samples contain mainly ellagitannins and lignans.

The metabolomics study revealed that sericic acid, sericoside and resveratrol-3-*O*-*β*-rutinoside are major constituents of *T. sericea* root bark, contrary to previous reports that limit the major constituents to sericoside. The root bark extracts displayed the best antibacterial activity towards *B. cereus*, a pathogen associated with food poisoning. Quercetin-3-(2′′-galloylrhamnoside) (**7**) displayed noteworthy activity against the pathogen with an MIC of 0.05 mg/mL. This aspect of the study, which validates the traditional use of the plant, should be further investigated. Flavogallonic acid dilactone and its methyl derivative, methyl-flavogallonate, were identified as the most active antibacterial compounds towards the range of pathogens tested. The variation in the activity of the extracts against *S. typhi* was associated with the concentration of sericic acid and resveratrol-3-*O*-*β*-rutinoside, two major yet inactive compounds. Despite a biochemometric study, no definite compounds could be pinpointed as being responsible for the antibacterial activity towards *S. typhi*, highlighting the complex interactions between the secondary compounds present. This study has revealed that a crude extract of *T. sericea* root bark with good antibacterial activity against *S. typhi* is characterised by a high concentration of flavogallonic acid dilactone and its derivative, and a low concentration of sericic acid and resveratrol-3-*O*-*β*-rutinoside. We therefore conclude that the major constituents of *T. sericea* root bark contribute to the chemical and antibacterial variation among different samples. They are of significance in the quality control of raw materials and products that exploit the antibacterial activity of this plant.

## 3. Materials and Methods

### 3.1. Sampling and Sample Preparation

Root bark samples from 42 individual trees were collected from 10 populations (labelled P1 to P10) across Limpopo Province, South Africa (Table 3), between May and June 2015. Twenty-five samples (six populations) were collected from Vhembe District, while nine samples (two populations) were collected from Mopani District. The remaining eight samples were collected from the Waterberg District. Composite samples were also prepared from the collected samples. The trees were identified by Prof Peter Tshisikhawe (Department of Botany, University of Venda, Thohoyandou, South Africa). For each population, a voucher specimen was prepared and deposited in the herbarium of the Department of Botany (voucher numbers are reported in Table 3). After debarking and washing to remove debris, the root bark was air-dried for two weeks and then ground to a fine powder using a grinder (Büchi Labortechnik, Flawil, Switzerland). A 5.0 g portion of each sample was soaked in 200 mL 1:1 dichloromethane:methanol (analytical grade, Merck, Germany) (1:1) for 24 h and filtered (Whatman No 4 filter paper). The filtrates were evaporated to dryness using a rotary evaporator (Buchi, Switzerland) at 45 °C. The extraction procedure was repeated twice, and the filtrates were combined. The dried crude extracts were weighed and stored at −20 °C.

### 3.2. UPLC-MS Analysis

The UPLC-MS analysis was carried out using a Waters Acquity Ultra Performance Liquid Chromatographic system with a Micromass–LCT Premier quadrupole time-of-flight mass spectrometer (QToF-MS) (Waters Corp., Milford, MA, USA) and a photodiode array (PDA) detector (Waters Corp., Milford, MA, USA) detector. An injection volume of 2.0 μL (full-loop injection) was used. Separation was achieved on an Acquity UPLC BEH C_18_ column (150 mm × 2.1 mm i.d., 1.7 μm particle size; Waters, Milford, MA, USA), maintained at 40 °C. The mobile phase consisted of 0.1% formic acid (Solvent A) and HPLC grade (Merck, Darmstadt, Germany) acetonitrile (Solvent B) at a flow rate of 0.3 mL/min. Gradient elution was executed as follows: the initial ratio was 10% B for 4 min, changed to 50% B for 6 min, then 95% B in 2.5 min, maintaining for 0.5 min, before returning to the initial ratio in 0.5 min. The system was equilibrated for 2 min prior to the subsequent analysis. Both positive and negative electrospray ionization (ESI) modes were evaluated, but the positive mode was selected for further sample analysis, since it resulted in a greater abundance of ions and provided information-rich spectra. A blank sample (methanol alone) and quality control (QC) samples (aliquots of all samples combined in one vial) were analysed to establish background peaks and assist with data alignment and instrument precision, respectively.

### 3.3. Chemometric Analysis of LC-MS Data

Raw data (114 samples by 763 compounds) matrix obtained from the UPLC-MS analysis were preprocessed using Masslynx software version 4.0 (Waters) and then exported to Microsoft Excel, and subsequently imported into MetaboAnalyst 4.0 (www.metaboanalyst.ca/MetaboAnalyst/home.xhtml), a free web-based software, designed for high-throughput metabolomic data analysis. Data were filtered (mean intensity) and normalised using quantile normalisation and pareto scaling to construct the best model, as determined from the model statistics obtained. According to the empirical rules based on the type of filtering used, 25% of the compounds were filtered, and the new matrix after data filtering was 114 samples by 572 compounds. A chemometric analysis of the UPLC-MS data was carried out following an untargeted approach using HCA on principal components. Chemotypes of the clusters obtained from HCA were identified by PLS-DA. A biochemometric analysis was carried out by aligning the UPLC-MS data with the antibacterial activities of the crude extracts. The UPLC-MS data were assigned to two classes based on the activities of the crude extracts. UPLC-MS data of extracts with higher activity (MICs < 1 mg/mL) were assigned to Class 1 (active), while UPLC-MS data of extracts with lower activity (MICs ≥ 1 mg/mL) were assigned to Class 2 (not active). An S-plot was constructed from the obtained OPLS-DA model to reveal compounds responsible for the separation of the two classes.

### 3.4. Isolation of Chemical Markers

Approximately 1.2 kg of the powdered bulk root bark was soaked in dichloromethane:methanol (1:1) for 48 h. The solvent was removed from the filtrate under reduced pressure at 40 °C to obtain 365 g of crude extract. A portion of the crude extract (140 g) was dissolved in methanol and adsorbed onto silica gel (Kieselgel 60, Merck). The dried sample was layered onto a silica gel column, which had been slurry-packed in ethyl acetate. Compounds were initially eluted with ethyl acetate, and finally with ethyl acetate:methanol (90:10) to obtain two corresponding fractions, namely Fraction 1 (18.56 g) and Fraction 2 (87.42 g).

A portion of Fraction 1 (12.6 g) was further purified using silica gel column chromatography (CC). The compounds were eluted with various mixtures of dichloromethane and methanol, by increasing the polarity step-wise, to yield four fractions (1a–d): 1a (2.6 g), 1b (0.10 g) and 1c (0.42 g) were eluted with dichloromethane:methanol (9:1), while 1d (5.8 g) was obtained with dichloromethane:methanol (7:3). Fraction 1a was further purified on silica gel by stepwise elution with hexane:ethyl acetate and ethyl acetate:methanol to obtain four fractions (1a1–4). Each fraction was analysed using UPLC-MS to identify the fractions containing peaks of interest (major peaks evident on the chemical profile). Fraction 1a2 contained a prominent compound (*m/z* 469). It was further purified by silica gel CC using gradient elution with mixtures of hexane, ethyl acetate and methanol to yield six fractions (1a2a-f). Of these, 1a2d (0.6 g) was further purified using preparative-high performance liquid chromatography-mass spectrometry (prep-HPLC-MS), to obtain compound (**1**) (342 mg, 0.04%).

A 60 g portion of Fraction 2 was subjected to column chromatography using Sephadex^®^ LH-20 (GE Healthcare, Danderyd, Sweden) as the stationary phase. Three fractions, 2a (7.30 g), 2b (20.28 g) and 2c (5.84 g) were obtained following elution with various ratios (9:1; 7:3 and 3:7) of dichloromethane:methanol. Since UPLC-MS analysis revealed that 2a contained the compounds of interest (*m/z* 536, *m/z* 469), it was further purified on silica gel using mixtures of hexane:ethyl acetate and ethyl acetate:methanol sequentially. The obtained fractions 2a1 and 2a2 were finally purified using targeted prep-HPLC-MS, to yield compounds (**2**) ([M + H]^+^ *m/z* 536, 238 mg) and (**3**) ([M + H]^+^ *m/z* 469, 500 mg). Compounds (**4**) ([M − H]^−^ *m/z* 301, 84 mg), (**5**) ([M − H]^−^ *m/z* 469, 100 mg), (**6**) ([M − H]^−^ *m/z* 483, 74 mg) (**7**) ([M − H]^−^ *m/z* 541, 17 mg), (**8**) ([M − H]^−^ *m/z* 599, 42 mg) were purified from Fraction 1d, while compound (**9**) ([M − H]^−^ *m/z* 695, 27 mg) was purified from Fraction 1c.

### 3.5. Purification Using Preparative-HPLC-MS

Final purification of the compounds was achieved using prep-HPLC-MS comprising an AutoPurification system interfaced with a QDa mass spectrometer (Waters, Milford, MA, USA). Conditions were optimised to achieve good resolution in a short analysis time. Partially purified fractions from the columns were prepared as 50 mg/mL solutions in methanol (HPLC grade, Merck) and introduced as 250 µL injection volumes. Separation was achieved on an XBridge Prep C_18_ column (250 mm × 19 mm i.d. 1.7 µm particle size, Waters) maintained at 40 °C. The mobile phase consisted of 0.1% formic acid in water (Solvent A) and methanol (Solvent B) at a flow rate of 20 mL/min. Gradient elution was performed as follows: the initial ratio was 10% B, held for 1 min, changed to 48% B within 1 min, changed to 65% B within 5 min, to 95% B within 5 min, maintaining for 1.5 min, before returning to the initial ratio in 0.5 min. Data were collected using MassLynx 4.1 (Waters, Milford Corp., MA, USA) chromatographic software. A probe temperature of 500 °C and a source temperature of 120 °C were maintained. The capillary and cone voltages were set to 800 and 10 V, respectively. Data were collected over the range *m/z* 100 to 750. The eluents containing the target compounds (*m/z* 535, 469, 711) were collected as 220 drops/tube (about 2 mL) using a fraction collector. Appropriately combined fractions were concentrated using a centrifugal evaporator (Genevac EZ 2 plus, Ipswic, UK) to yield residues, which were subsequently analysed by UPLC-MS as described.

### 3.6. Nuclear Magnetic Resonance (NMR) Spectroscopy

One- and bi-dimensional NMR experiments were carried out using a Bruker Ultra ShieldTM Plus 400 MHz (Biospin) spectrometer (Bruker, Bellericea, MA, USA). Recorded spectra were processed using Bruker Topspin 3.2 on the AVIII 400 software. Compounds (10 mg) were dissolved in 0.7 mL of deuterated methanol (Methanol-d4) and transferred to NMR tubes before analysis.

### 3.7. Evaluation of the Antibacterial Activity

Pathogens selected for this study were the Gram-positive bacteria, *Bacillus cereus* (ATCC 11778), *Staphylococcus epidermidis* (ATCC 12223) and *Staphylococcus aureus* (ATCC 25923), while the Gram-negative bacteria included *Escherichia coli* (ATCC 8739), *Shigella sonnei* (ATCC 9292), *Pseudomonas aeruginosa* (ATCC 27853), *Klebsiella pneumoniae* (ATCC 13883) and *Salmonella typhimurium* (*S. typhi*, ATCC 14028). Selection of these test micro-organisms were based on pathogenesis as reflected by traditional use i.e., *K. pneumoniae* (respiratory pathogen), *S. epidermidis, S. aureus* and *P. aeruginosa* (skin pathogens), and *E. coli, S. sonnei* and *S. typhimurium* (gastro-intestinal pathogens).

Crude extracts (dichloromethane:methanol 1:1) prepared from the 10 populations were used against two pathogens (*B. cereus* and *S. typhi*) to determine the possible effect of geographical variation on the antibacterial activity and to correlate the phytochemistry to the activity of the *T. sericea* root bark. Stock solutions of the crude extracts were prepared at a concentration of 32 mg/mL, while the pure compounds were prepared at 5.0 mg/mL for the antibacterial assays. The samples were dissolved in either dimethyl sulfoxide (DMSO) or acetone (both AR grade; Sigma Aldrich, St. Louis, MI, USA) depending on the solubility of the samples or compounds in the solvents where final concentration did not exceed 5% in the first well of the microtitre plate.

The serial dilution assay was used to determine the antibacterial activity of each dichloromethane:methanol (1:1) extract as described by Eloff [46] with modifications as described by van Vuuren et al. [18]. Cultures were inoculated in Tryptone Soya broth (TSB) and incubated at 37 °C for 24 h to prepare overnight cultures for the assay. Microplates were aseptically prepared by placing 100 µL sterile broth in each well. Thereafter, 100 µL of the individual test samples (32 or 5.0 mg/mL) was added to the first row of each plate. Serial dilutions were performed by removing 100 μL of the mixed extract and broth from each well and transferring to the well in the next row, resulting in a final volume of 100 µL in each well. Ciprofloxacin (0.01 mg/mL) was used as the positive control, while acetone or DMSO were used as negative controls. A culture control (broth with culture) was included to monitor viability and response time to indicator.

The overnight culture of each pathogen was diluted with fresh sterile TSB at a 1:100 ratio to provide an approximate inoculum size of 1 × 10^6^ colony-forming units (CFU)/mL. The inoculum size was estimated by comparing the turbidity of the inoculum visually with a 0.5 McFarland standard solution. A 100 µL volume of the culture was added to each well and the plates which were covered with sterile adhesive film. They were then incubated at 37 °C for 24 h (EcoTherm, Hartkirchen, Austria). After incubation, 40 µL of 0.2 mg/mL *p*-iodonitrotetrazolium violet (Sigma-Aldrich, St Louis, MI, USA) was added to the incubated wells. The plates were maintained at ambient temperature for time periods 2–6 h depending on the response time of indicator to bacterial growth of each pathogen Readings were taken once there was change of colour of the culture control (growth of pathogen without inhibitor) to pink. The experiments were carried out in duplicate.

## 4. Conclusions

This study has revealed chemotypic variation that is not population-specific, within root bark samples. Three groups, characterised by high concentrations of sericic acid, an unidentified triterpenoid glycoside and sericoside, respectively, were identified, suggesting that even more variation may exist amongst natural populations. Although the activities recorded against a range of gastro-intestinal and respiratory pathogens were in most cases not noteworthy, the broad-spectrum antibacterial activity of individual compounds (flavogallonic acid dilactone and methyl flavogallonate) deserves further attention. Throughout the study it became clear that a delicate balance between the active and non-active compounds strongly influences the activities of the crude extract. This aspect will have to be further investigated using pure compounds, to determine additive, synergistic or attenuating interactions between the compounds, if root bark extracts are to be fully commercialised.

## Figures and Tables

**Table 1:** Minimum inhibitory concentrations (MICs, in mg/mL) indicating the antibacterial activities of crude dichloromethane:methanol extracts, fractions and pure compounds from *T. sericea* against a range of bacterial pathogens.

Samples	*B.c*	*S.a*	*S.e*	*E.c*	*K.p*	*P.a*	*S.s*	*S.t*
CE	0.29	1.50	1.50	1.50	3.00	0.75	1.50	1.50
F1 F2	0.29 >3.00	0.75 3.00	0.75 1.50	1.50 >3.0	1.50 3.00	0.75 1.50	1.50 1.50	1.50 1.50
(1)	0.38	0.75	0.75	0.38	0.38	0.38	0.29	>0.75
(2)	>1.30	>1.30	>1.30	>1.30	>1.30	>1.30	>1.30	>1.30
(3)	>1.30	>1.30	>1.30	>1.30	1.30	>1.30	>1.30	>1.30
(4)	0.22	0.75	0.75	0.38	>0.75	0.38	0.57	0.38
(5)	0.11	0.50	0.75	0.38	0.38	0.38	0.75	0.25
(6)	0.12	0.75	0.75	0.38	0.38	0.38	0.38	0.32
(7)	0.05	NT	0.75	0.38	>0.75	NT	0.38	0.38
(9)	0.14	NT	>0.75	0.80	>0.75	NT	0.38	0.75
Cipro	0.04	0.08	0.08	1.25	0.08	0.08	1.25	0.04

*B.c*: *Bacillus cereus* (ATCC 11778), *S.a*: *Staphylococcus aureus* (ATCC 25923), *S.e*: *Staphylococcus epidermidis* (ATCC 12223), *E.c*: *Escherichia coli* (ATCC 8739), *K.p*: *Klebsiella pneumoniae* (ATCC 13883), *P.a*: *Pseudomonas aeruginosa* (ATCC 27853), *S.s*: *Shigella sonnei* (ATCC 9292), *S.t*: *Salmonella typhimurium* (ATCC 14028). CE: Crude extract, F1: Fraction 1, (1): sericic acid, (2): sericoside, (3): resveratrol-3-*O*-*β*-rutinoside, (4): ellagic acid, (5): flavogallonic acid dilactone, (6): methyl-(S)-flavogallonate, (7): quercetin-3-(2′′- galloylrhamnoside, (9): arjunetin, Cipro: Ciprofloxacin (positive control in μg/mL). NT: Not tested (insufficient compound yield).

**Table 2:** Variation in antibacterial activities as reflected by the MIC (mg/mL) of dichloromethane:methanol extracts of *T. sericea* root bark collected from different populations.

Population	*B. cereus*	*S. typhi*
P1.1	1.0	0.50
P1.2	1.0	0.25
P1.3	1.0	0.25
P2.1	1.0	0.25
P2.2	1.0	0.25
P2.3	1.0	0.50
P2.4	1.0	0.50
P3.1	1.0	0.25
P3.2	1.0	0.25
P3.3	1.0	0.25
P3.4	1.0	0.25
P4.1	1.0	1.0
P4.2	1.0	1.0
P4.3	1.0	1.0
P4.4	1.0	1.0
P4.5	1.0	1.0
P5.1	1.0	1.0
P5.2	1.0	1.0
P5.3	1.0	1.0
P5.4	1.0	1.0
P6.1	1.0	1.0
P6.2	1.0	1.0
P6.3	1.0	1.0
P6.4	1.0	1.0
P6.5	1.0	1.0
P7.1	1.0	1.0
P7.2	0.75	0.50
P7.3	1.0	0.50
P7.4	1.0	0.50
P7.5	1.0	0.50
P8.1	1.0	0.50
P8.2	1.0	0.50
P8.3	1.0	0.50
P8.4	1.0	0.50
P8.5	1.0	0.50
P9.1	1.0	0.50
P9.2	1.0	0.50
P9.3	1.0	0.50
P9.4	1.0	0.50
P9.5	1.0	0.25
P10.1	1.0	0.50
P10.2 Cipro *	1.0 0.04	0.50 0.04

Cipro *: Ciprofloxacin (positive control in μg/mL). P: Population.

**Table 3:** Coordinates of the locations where samples were collected and the corresponding voucher specimen numbers.

Popu-lation	Code	Location	District	Coordinates	No of Samples	Voucher No
	BP	Bela-Bela/ Pretoria (N1)	Waterberg	S24°47′51.9″ E28°27′03.6″	3	CPA001
P2	G	Muyexe, Giyani	Mokopani	S23°11′22.6″ E30°55′05.3″	4	CPA002
	J	Maila, close to the N1	Vhembe	S23°14′47.0″ E29°53′06.8″	4	CPA003
P4	K	Along Punda Maria/Kruger road	Vhembe	S22°58′22.0″ E30°27′27.0″	5	CPA004
P5	MM	Mavambe, Malamulele	Vhembe	S23°00′02.1″ E30°39′09.0″	4	CPA005
P6	MP	Mokopong/Pretoria along N1	Waterberg	S24°20′41.6″ E28°53′47.4″	5	CPA006
P7	TSA	Tshandama, Thengwe	Vhembe	S22°45′43.0″ E30°30′34.3″	5	CPA007
P8	TSH	Tshitavha, Sambandou	Vhembe	S22°44′41.2″ E30°38′41.2″	5	CPA008
P9	TZ	Modjadjiskloof, along Tzaneen road	Mokopani	S23°33′14.4″ E30°03′55.6″	5	CPA009
P10	V	Vuwani	Vhembe	S23°07′46.8″ E30°22′46.9″	2	CPA010

## Supplementary Materials

The following are available online. Figure S1: Chemical profile of selected *T. sericea* root bark samples, Figure S2: ^1^H NMR spectrum of sericic acid, Figure S3: ^13^C NMR spectrum of sericoside, Figure S4: ^1^H NMR, ^13^C NMR spectra of 3′,5′,4–trihydroxy-resveratrol-3-*O*-*β*-rutinoside, Figure S5: ^1^H NMR, ^13^C NMR spectra of ellagic acid, Figure S6: ^1^H NMR spectrum of flavogallonic acid dilactone, ^1^H NMR of spectrum of methyl-flavogallonate C) ^13^C NMR spectrum of flavogallonic acid dilactonetitle, Figure S7: ^1^H NMR, ^13^C NMR spectra of resveratrol 3-(6′′-galloyl)-*O*-*β*-d-glucopyranoside, Figure S8: ^1^H NMR, ^13^C NMR, UPLC-MS^2^ spectra of quercetin-3-(2′′-galloylrhamnoside), Figure S9: ^1^H NMR, ^13^C NMR, UPLC-MS^2^ spectra of arjunetin, Figure S10: OPLS-DA model of the active and non-active samples of *T. sericea* root bark, Figure S11: Variable importance for project (VIP) scores of compounds associated with the antibacterial activities of the active and non-active classes.

## References

[1] Moshi M.J., Mbwambo Z.H. (2005). Some pharmacological properties of extracts of *Terminalia sericea* roots. J. Ethnopharmacol..

[2] Likoswe M.G., Njoloma J.P., Mwase W.F., Chilima C.Z. (2008). Effect of seed collection times and pretreatment methods on germination of *Terminalia sericea* Burch. ex DC. Afr. J. Biotechnol..

[3] Chivandi E., Davidson B., Erlwanger K. (2013). Proximate, mineral, fibre, phytate–phosphate, vitamin E, amino acid and fatty acid composition of *Terminalia sericea*. S. Afr. J. Bot..

[4] van Wyk B.-E., van Oudtshoorn B., Gericke N. (2013). Medicinal Plants of South Africa.

[5] Rukangira E. (2001). Medicinal plants and traditional medicine in Africa: Constraints and challenges. Sustain. Dev. Int..

[6] Rode T. (2003). Complex formation of sericoside with hydrophilic cyclodextrins: Improvement of solubility and skin penetration in topical emulsion based formulations. Eur. J. Pharm. Biopharm..

[7] Pagin I., Togni S., Maramaldi G., Cattaneo R., Caccia G., Eggenhoffner R., Giacomelli L. (2016). Anti-aging effects of a novel sericoside 0.5% cream in reducing skin wrinkles and ameliorating skin texture. Dermatol. Exp..

[8] Katerere D.R., Gray A.I., Nash R.J., Waigh R.D. (2012). Phytochemical and antimicrobial investigations of stilbenoids and flavonoids isolated from three species of Combretaceae. Fitoterapia.

[9] Eloff J.N. (1999). The antibacterial activity of 27 southern African members of the Combretaceae. S. Afr. J. Sci..

[10] Fyhrquist P., Mwasumbi L., Hæggström C.-A., Vuorela H., Hiltunen R., Vuorela P. (2002). Ethnobotanical and antimicrobial investigation on some species of *Terminalia* and *Combretum* (Combretaceae) growing in Tanzania. J. Ethnopharmacol..

[11] Steenkamp V., Mathivha E., Gouws M., Van Rensburg C. (2004). Studies on antibacterial, antioxidant and fibroblast growth stimulation of wound healing remedies from South Africa. J. Ethnopharmacol..

[12] Eldeen I.M., Elgorashi E.E., van Staden J. (2005). Antibacterial, anti-inflammatory, anti-cholinesterase and mutagenic effects of extracts obtained from some trees used in South African traditional medicine. J. Ethnopharmacol..

[13] Tshikalange T., Meyer J., Hussein A. (2005). Antimicrobial activity, toxicity and the isolation of a bioactive compound from plants used to treat sexually transmitted diseases. J. Ethnopharmacol..

[14] York T., van Vuuren S.F., de Wet H. (2012). An antimicrobial evaluation of plants used for the treatment of respiratory infections in rural Maputaland, KwaZulu-Natal, South Africa. J. Ethnopharmacol..

[15] Mabona U., Van Vuuren S.F. (2013). Southern African medicinal plants used to treat skin diseases. S. Afr. J. Bot..

[16] Cock I.E., van Vuuren S.F. (2014). Anti-Proteus activity of some South African medicinal plants: Their potential for the prevention of rheumatoid arthritis. Inflammopharmacology.

[17] Cock I.E., van Vuuren S.F. (2015). The potential of selected South African plants with anti-Klebsiella activity for the treatment and prevention of ankylosing spondylitis. Inflammopharmacology.

[18] van Vuuren S.F., Nkwanyana M.N., de Wet H. (2015). Antimicrobial evaluation of plants used for the treatment of diarrhoea in a rural community in northern Maputaland, KwaZulu-Natal, South Africa. BMC Complement. Altern. Med..

[19] Netshiluvhi T., Eloff J. (2016). Influence of annual rainfall on antibacterial activity of acetone leaf extracts of selected medicinal trees. S. Afr. J. Bot..

[20] Eldeen I.M., Elgorashi E.E., Mulholland D.A., van Staden J. (2006). Anolignan B: A bioactive compound from the roots of *Terminalia sericea*. J. Ethnopharmacol..

[21] Eldeen I.M., Van Heerden F.R., Van Staden J. (2008). Isolation and biological activities of termilignan B and arjunic acid from *Terminalia sericea* roots. Planta Med..

[22] Ulrich-Merzenich G.S. (2014). Combination screening of synthetic drugs and plant derived natural products—Potential and challenges for drug development. Synergy.

[23] Faruque M.O., Ankhi U.R., Kamaruzzaman M., Barlow J.W., Zhou B., Hao J., Yang X., Hu X. (2019). Chemical composition and antimicrobial activity of *Congea tomentosa*, an ethnomedicinal plant from Bangladesh. J. Ind. Crop. Prod..

[24] Abdelgaleil S., Saad M., Ariefta N., Shiono Y. (2020). Antimicrobial and phytotoxic activities of secondary metabolites from *Haplophyllum tuberculatum* and *Chrysanthemum coronarium*. S. Afr. J. Bot..

[25] Wink M. (2003). Evolution of secondary metabolites from an ecological and molecular phylogenetic perspective. J. Phytochem..

[26] Bombardelli E., Bonati A., Gabetta B., Mustich G. (1974). Triterpenoids of *Terminalia sericea*. Phytochemistry.

[27] Bombardelli E., Martinelli E., Mustich G. (1975). Plants of Mozambique. IX. A new hydroxystilbene glycoside from *Terminalia sericea*. Fitoterapia.

[28] Joseph C.C., Moshi M., Innocent E., Nkunya M. (2007). Isolation of a stilbene glycoside and other constituents of *Terminalia sericeae*. Afr. J. Tradit. Complement. Altern. Med..

[29] Hess S.C., Monache F.D. (1999). Divergioic acid, a triterpene from *Vochysia divergens*. J. Braz. Chem. Soc..

[30] Rahman A.-u., Zareen S., Choudhary M.I., Akhtar M.N., Ngounou F. (2005). Some chemical constituents of *Terminalia glaucescens* and their enzymes inhibition activity. Z. Naturforsch. C J. Biosci..

[31] Tchuenmogne T., Aimée M., Kammalac T.N., Gohlke S., Kouipou R.M.T., Aslan A., Kuzu M., Comakli V., Demirdag R., Ngouela S.A. (2017). Compounds from *Terminalia mantaly* L.(Combretaceae) stem bark exhibit potent inhibition against some pathogenic yeasts and enzymes of metabolic significance. Medicines.

[32] Asres K., Bucar F., Edelsbrunner S., Kartnig T., Höger G., Thiel W. (2001). Investigations on antimycobacterial activity of some Ethiopian medicinal plants. Phytother. Res..

[33] Gossan D.P.A., Magid A.A., Yao-Kouassi P.A., Josse J., Gangloff S.C., Morjani H., Voutquenne-Nazabadioko L. (2016). Antibacterial and cytotoxic triterpenoids from the roots of *Combretum racemosum*. Fitoterapia.

[34] Wanjala C.C., Majinda R.R. (2001). A new stilbene glycoside from *Elephantorrhiza goetzei*. Fitoterapia.

[35] Kim J.-P., Lee I.-K., Yun B.-S., Chung S.-H., Shim G.-S., Koshino H., Yoo I.-D. (2001). Ellagic acid rhamnosides from the stem bark of *Eucalyptus globulus*. Phytochemistry.

[36] Srivastava A., Jagan Mohan Rao L., Shivanandappa T. (2007). Isolation of ellagic acid from the aqueous extract of the roots of *Decalepis hamiltonii*: Antioxidant activity and cytoprotective effect. Food Chem..

[37] Orabi M.A., Yoshimura M., Amakura Y., Hatano T. (2015). Ellagitannins, gallotannins, and gallo-ellagitannins from the galls of *Tamarix aphylla*. Fitoterapia.

[38] Mohieldin E.A.M., Muddathir A.M., Yamauchi K., Mitsunaga T. (2017). Anti-caries activity of selected Sudanese medicinal plants with emphasis on *Terminalia laxiflora*. Rev. Bras. Farmacogn..

[39] Salih E.Y., Fyhrquist P., Abdalla A., Abdelgadir A.Y., Kanninen M., Sipi M., Luukkanen O., Fahmi M.K., Elamin M.H., Ali H.A.J.A. (2017). LC-MS/MS tandem mass spectrometry for analysis of phenolic compounds and pentacyclic triterpenes in antifungal extracts of Terminalia brownii (Fresen). Antibiotics.

[40] Marzouk M.S., El-Toumy S.A., Moharram F.A. (2002). Pharmacologically active ellagitannins from *Terminalia myriocarpa*. Planta Med..

[41] Okasaka M., Takaishi Y., Kogure K., Fukuzawa K., Shibata H., Higuti T., Honda G., Ito M., Kodzhimatov O.K., Ashurmetov O. (2004). New stilbene derivatives from *Calligonum leucocladum*. J. Nat. Prod..

[42] Estrada O., Hasegawa M., Gonzalez-Mujica F., Motta N., Perdomo E., Solorzano A., Mendez J., Mendez B., Zea E.G. (2005). Evaluation of flavonoids from *Bauhinia megalandra* leaves as inhibitors of glucose-6-phosphatase system. Phytother. Res..

[43] Bentley J., Moore J.P., Farrant J.M. (2019). Metabolomic profiling of the desiccation-tolerant medicinal shrub *Myrothamnus flabellifolia* indicates phenolic variability across its natural habitat: Implications for tea and cosmetics production. Molecules.

[44] Singh D., Gupta M., Tripathi A., Prajapati V., Kumar S. (2004). Arjunetin from *Terminalia arjuna* as an insect feeding-deterrent and growth inhibitor. Phytother. Res..

[45] Salih E.Y.A., Kanninen M., Sipi M., Luukkanen O., Hiltunen R., Vuorela H., Julkunen-Tiitto R., Fyhrquist P. (2017). Tannins, flavonoids and stilbenes in extracts of African savanna woodland trees *Terminalia brownii*, *Terminalia laxiflora* and *Anogeissus leiocarpus* showing promising antibacterial potential. S. Afr. J. Bot..

[46] Eloff J. (1998). Which extractant should be used for the screening and isolation of antimicrobial components from plants?. J. Ethnopharmacol..

