# Supplementary material for: Unravelling the Antibacterial Activity of Terminalia sericea Root Bark through a Metabolomic Approach
Source: Molecules. 2020 Aug 13;25(16):3683. doi: 10.3390/molecules25163683 (PMC7464275; doi:10.3390/molecules25163683)
Supplement: Supplementary file 1 [file molecules-25-03683-s001.pdf]

SUPPLEMENTARY MATERIAL

Type of the Paper (Article)

Unraveling the antibacterial activity of *Terminalia sericea* root bark through a metabolomic approach

Chinedu P Anokwuru [1,2], Sidonie Tankeu [2], Sandy van Vuuren [3], Alvaro Viljoen [2,4]. Isaiah D.I Ramaite[1], Orazio Taglialatela-Scafati [5,1], Sandra Combrinck [2*]

[1] Department of Chemistry, University of Venda, Private Bag X5050, Thohoyandou 0950, South Africa Affiliation 1; anokwuruchi@gmail.com; Isaiah.Ramaite@univen.ac.za

[2] Department of Pharmaceutical Sciences, Tshwane University of Technology, Private Bag X680, Pretoria, 001, South Africa; anokwurucp@tut.ac.za; tankeus@tut.ac.za; combrincks@tut.ac.za

[3] Department of Pharmacy and Pharmacology, Faculty of Health Sciences, University of the Witwatersrand, 7 York Road, Parktown, 2193, South Africa; sandy.vanvuuren@wits.ac.za

[4] SAMRC Herbal Drugs Research Unit, Faculty of Science, Tshwane University of Technology, Private Bag X680, Pretoria 0001, South Africa; viljoenam@tut.ac.za

[5] Department of Pharmacy, University of Naples, Federico II Via D. Montesano 49, 1-80131 Napoli, Italy; scatagli@unina.it

* Correspondence: combrincks@tut.ac.za

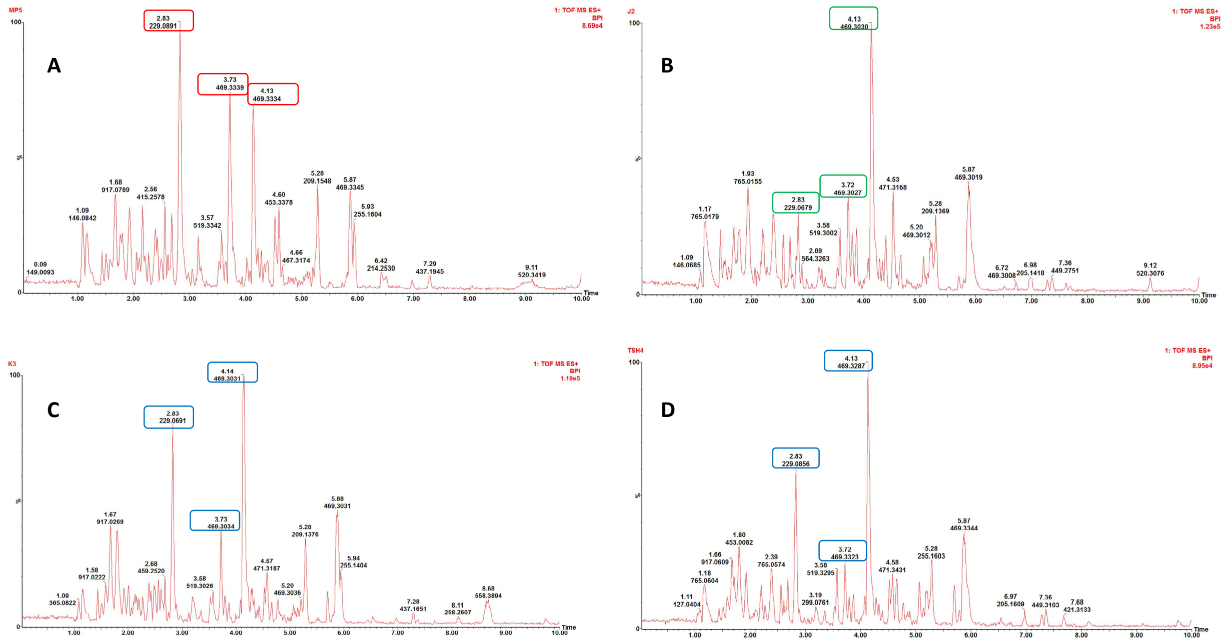

Figure S1: Chemical profile of selected *T. sericea* root bark samples from population A) P6, B) P3, C) P4, D) P8. Peaks of each chromatogram highlighted are major constituents clustered in branch X (red), Y (green) and Z (blue)

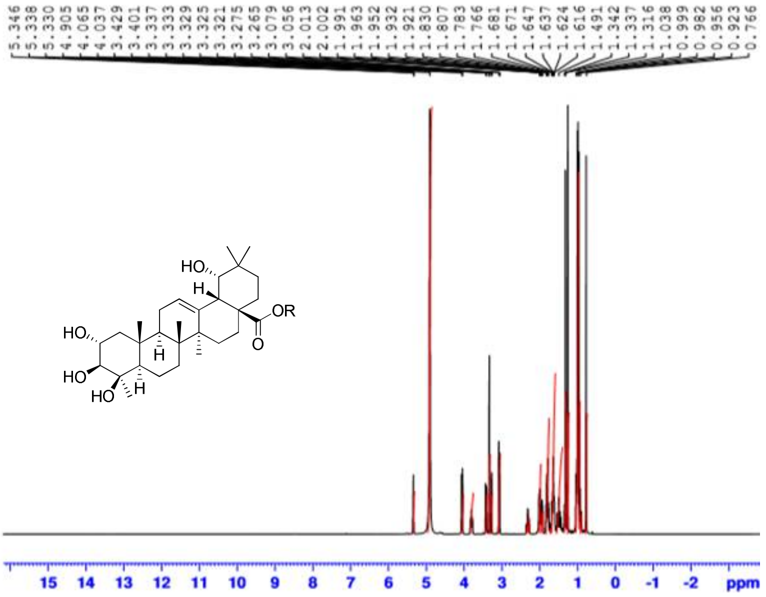

Figure S2: ^{1}H NMR spectrum of sericic acid

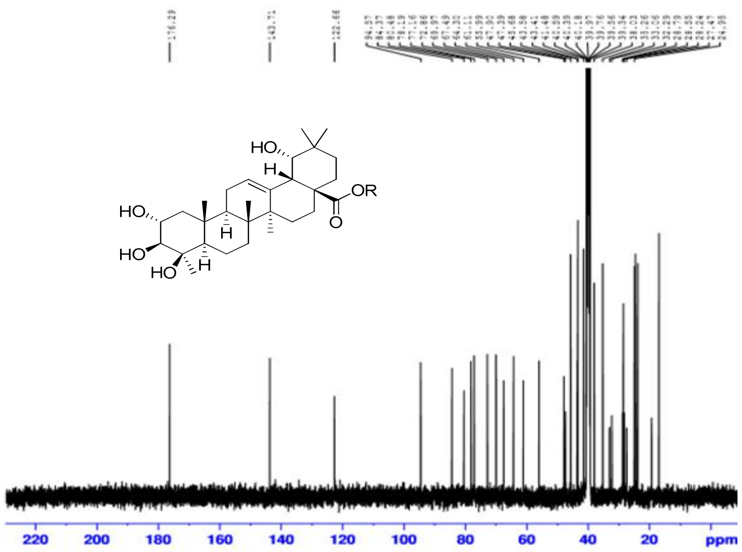

Figure S3: ^{13}C NMR spectrum of sericoside

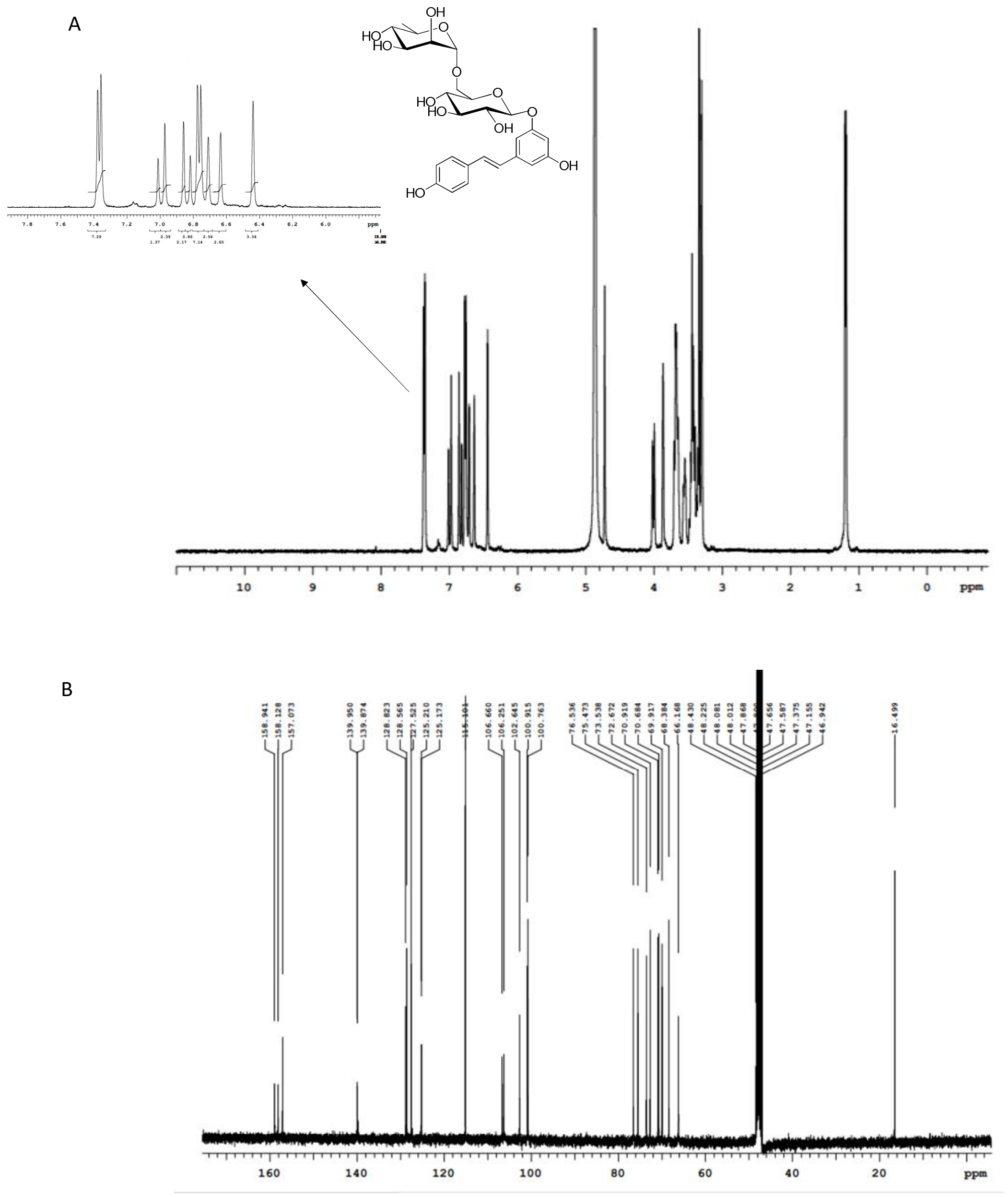

Figure S4: A) ^{1}H NMR , B) ^{13}C NMR spectra of 3',5',4–trihydroxy-resveratrol-3-*O*-*β*-rutinoside

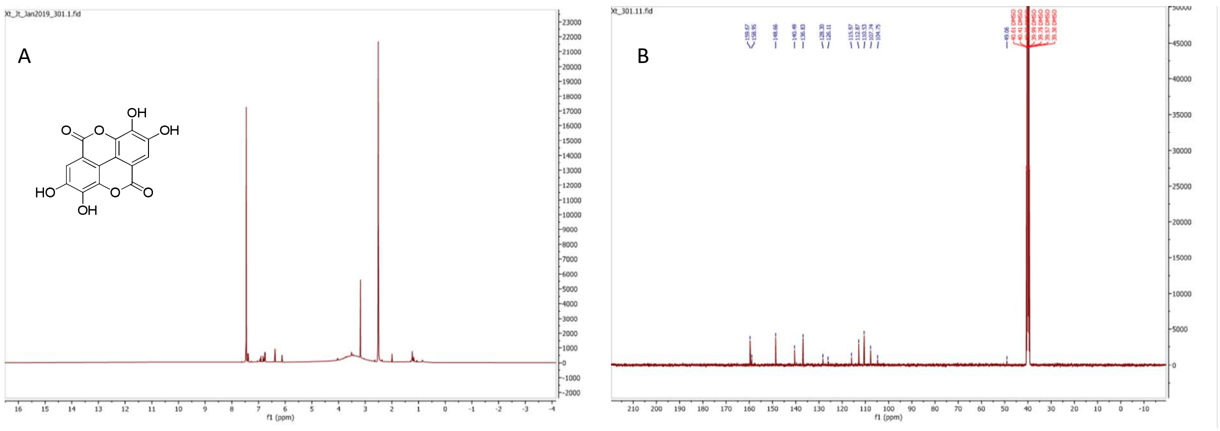

Figure S5: A) ^{1}H NMR , B) ^{13}C NMR spectra of ellagic acid

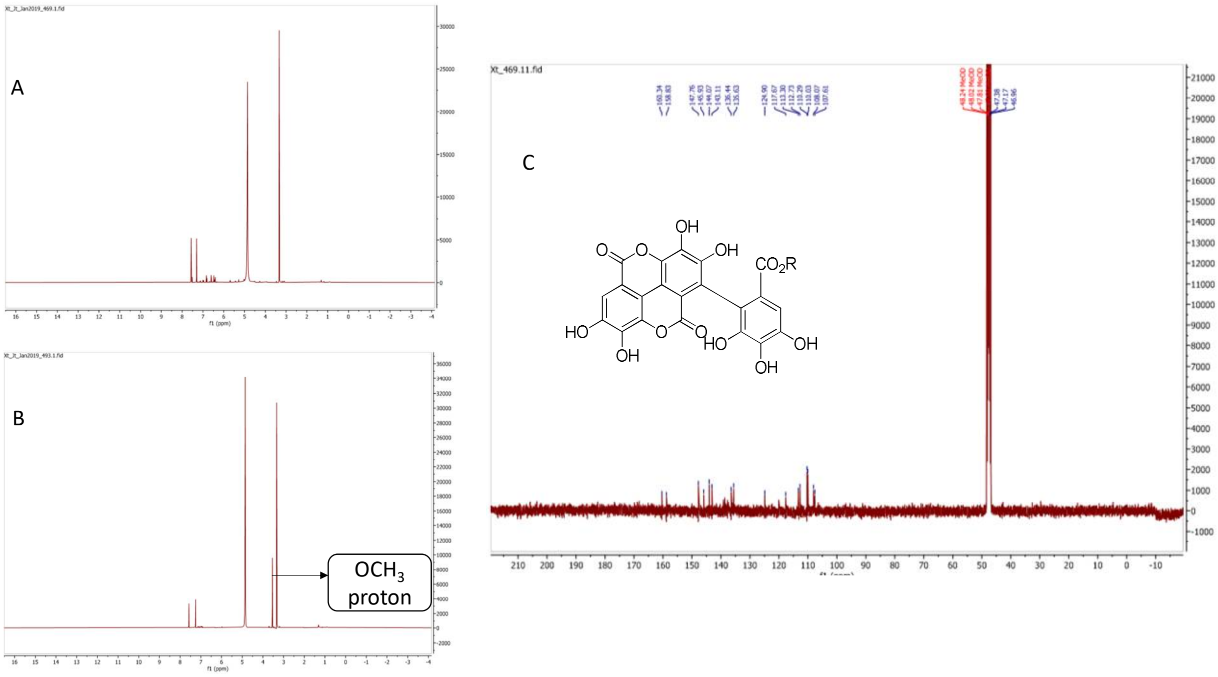

Figure S6: A) ^{1}H NMR spectrum of flavogallonic acid dilactone, B) ^{1}H NMR of spectrum of methyl-flavogallonate. The methoxy proton (OCH_3) at δ_H 3.4 confirms the presence of a methyl group. C) ^{13}C NMR spectrum of flavogallonic acid dilactone

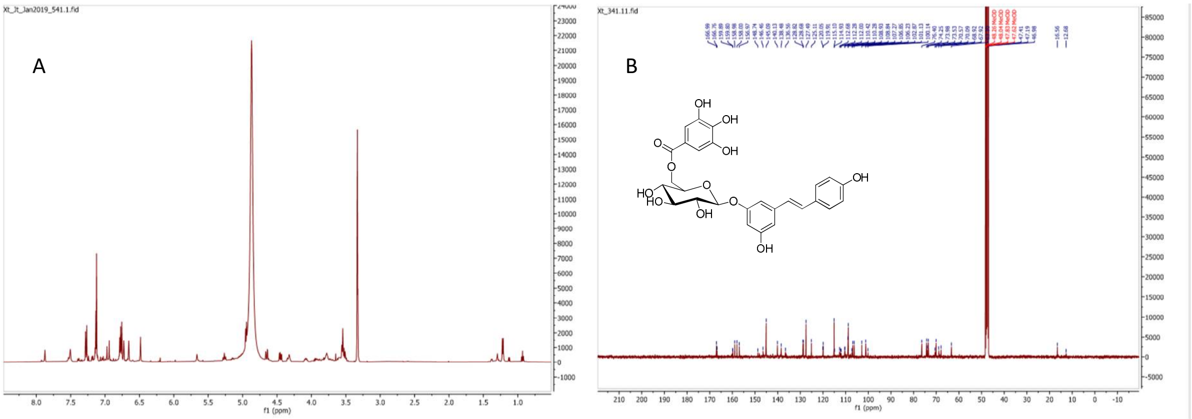

Figure S7: A) ^{1}H and B) ^{13}C NMR spectra of Resveratrol 3-(6″-galloyl)-*O*-*β*-D-glucopyranoside

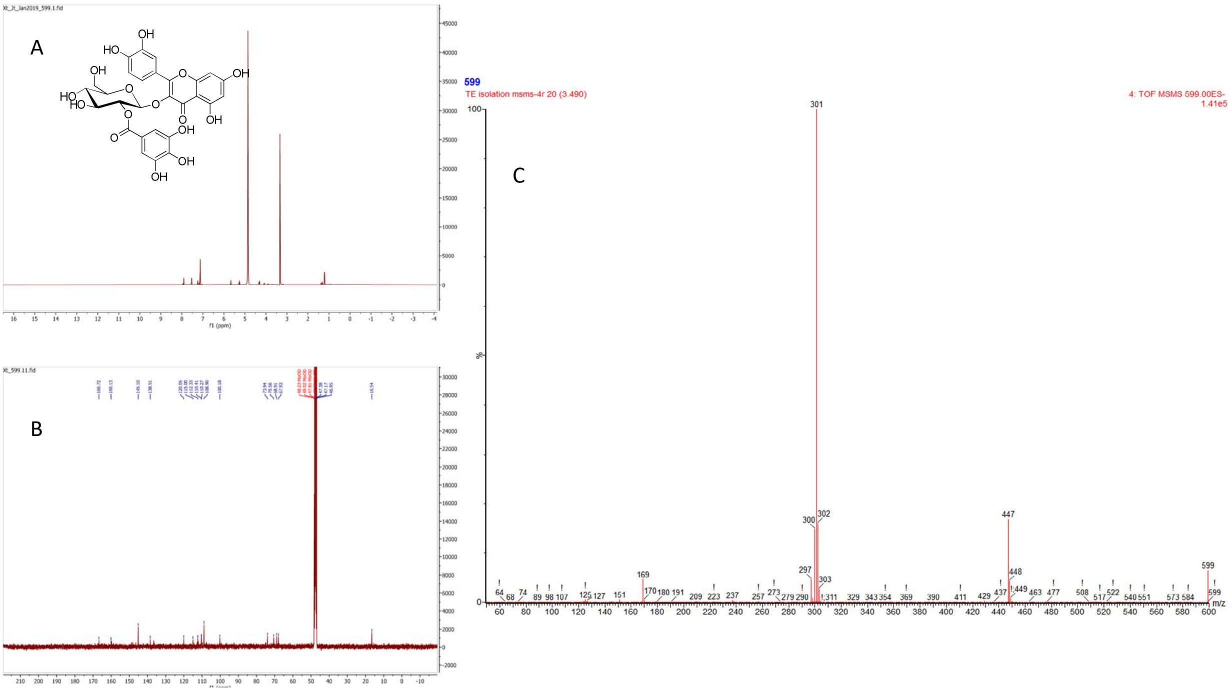

Figure S8: A) ^{1}H NMR , B) ^{13}C NMR spectra and C) UPLC-MS^{2} fragment ions of quercetin-3-(2″-galloylrhamnoside)

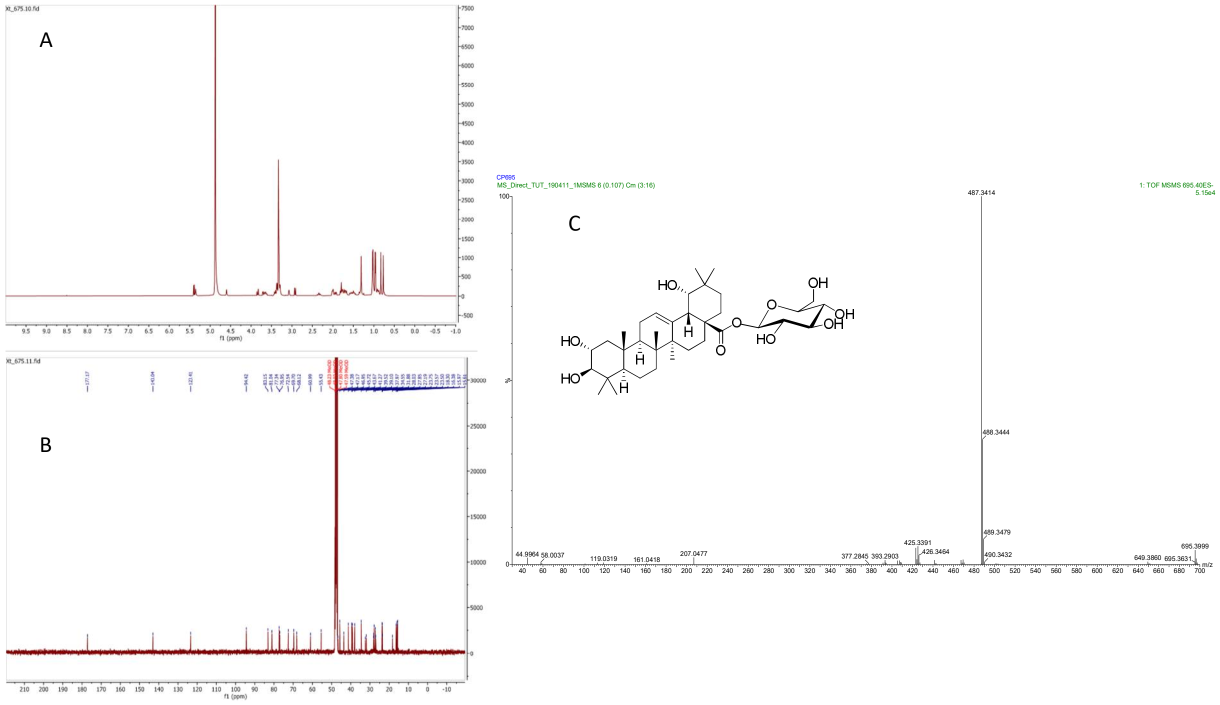

Figure S9: A) ^{1}H NMR , B) ^{13}C NMR spectra and C) UPLC-MS[2] fragment ions of ajunetin

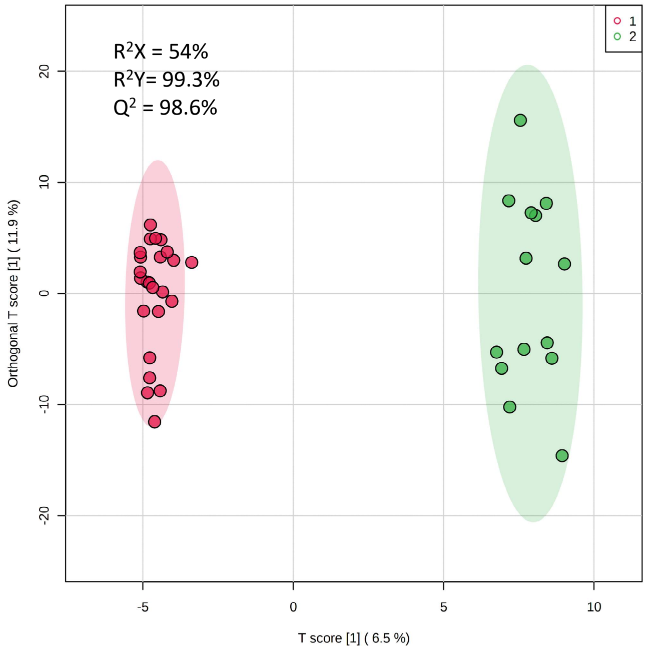

Figure S10: Orthogonal projection to latent structure-discriminant analysis (OPLS-DA) model of the active (RED) and non-active (GREEN) samples of *T. sericea* root bark.

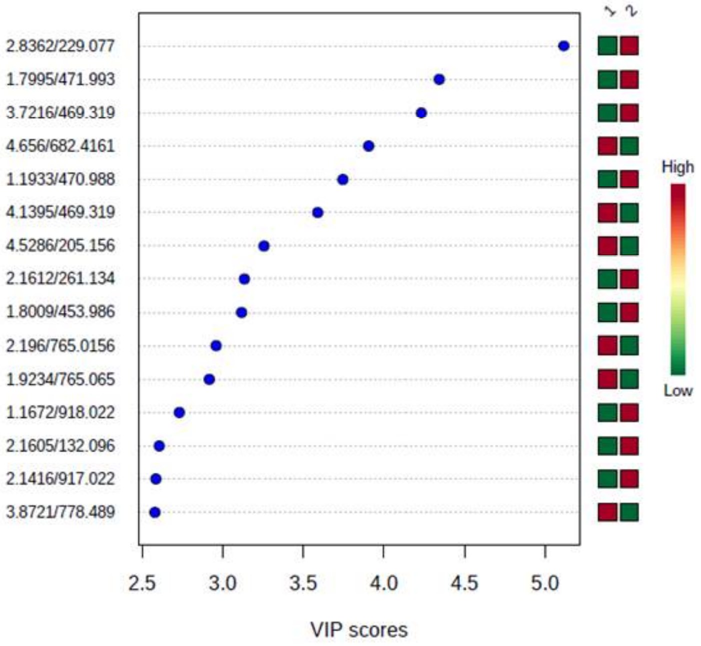

Figure S11: Variable importance for project (VIP) scores of compounds associated with the antibacterial activities of the active and non-active classes
